# Targeted Therapy in Acute Lymphoblastic Leukaemia

**DOI:** 10.3390/jpm11080715

**Published:** 2021-07-25

**Authors:** Ross Salvaris, Pasquale Luke Fedele

**Affiliations:** 1 Department of Clinical Haematology, Monash Health, Clayton 3168, Australia; ross.salvaris@monashhealth.org; 2 School of Clinical Sciences at Monash Health, Monash University, Clayton 3168, Australia

**Keywords:** acute lymphoblastic leukaemia, targeted therapy

## Abstract

The last decade has seen a significant leap in our understanding of the wide range of genetic lesions underpinning acute lymphoblastic leukaemia (ALL). Next generation sequencing has led to the identification of driver mutations with significant implications on prognosis and has defined entities such as BCR-ABL-like ALL, where targeted therapies such as tyrosine kinase inhibitors (TKIs) and JAK inhibitors may play a role in its treatment. In Philadelphia positive ALL, the introduction of TKIs into frontline treatment regimens has already transformed patient outcomes. In B-ALL, agents targeting surface receptors CD19, CD20 and CD22, including monoclonal antibodies, bispecific T cell engagers, antibody drug conjugates and chimeric antigen receptor (CAR) T cells, have shown significant activity but come with unique toxicities and have implications for how treatment is sequenced. Advances in T-ALL have lagged behind those seen in B-ALL. However, agents such as nelarabine, bortezomib and CAR T cell therapy targeting T cell antigens have been examined with promising results seen. As our understanding of disease biology in ALL grows, as does our ability to target pathways such as apoptosis, through BH3 mimetics, chemokines and epigenetic regulators. This review aims to highlight a range of available and emerging targeted therapeutics in ALL, to explore their mechanisms of action and to discuss the current evidence for their use.

## 1. Introduction

Acute lymphoblastic leukaemia (ALL) is a rare malignancy of committed B- or T-cell progenitors. There is a bimodal distribution with one peak in childhood, where it represents the most common paediatric malignancy, and a second peak around 50 years of age. Childhood ALL is one of the success stories of traditional chemotherapy and rigorous clinical trial methodology leading to cure rates now exceeding 90%. The outcomes for adult patients with ALL, however, are far inferior with an ongoing decline in survival with increasing age from adolescence [1]. The prognosis for patients who relapse, irrespective of age, is poor.

Over the last decade, technologies such as next-generation sequencing have helped to identify a wide range of genetic lesions that underpin ALL. An understanding of their prognostic significance has led to better patient risk stratification with an increasing opportunity for more personalised and targeted therapy. Concurrently, multiple novel therapies targeting vulnerabilities based on the underlying cell of origin or specific genetic drivers have become available, with many more now entering clinical trials. This review aims to highlight these available and emerging targeted therapeutics in ALL, to explore their mechanisms of action and to discuss the current evidence for their use. 

## 2. Impact of Next-Generation Sequencing Technologies on the Classification of ALL

Traditionally the classification of ALL has been centred upon assessment of the immunophenotype, based on the expression of either B- or T-cell surface and intracellular proteins, and genetic alterations identified on karyotype and fluorescence in-situ hybridization (FISH). These cytogenetic tests can identify changes in chromosomal number, termed aneuploidy, as well as chromosomal rearrangement, such as the reciprocal translocation between chromosomes 9 and 22, t(9;22), which defines Philadelphia positive (Ph+) ALL. Some of the abnormalities identified on karyotype or FISH analysis reveal abnormalities that define a group of patients where a targeted therapy may be used. The use of tyrosine kinase inhibitors in Ph+ ALL, the first true class of targeted agents in this disease, is the best-known example of this. Furthermore, these genetic changes have prognostic significance and help to risk stratify patients. 

Whilst these traditional genetic tests continue to play an important role in the workup of patients with ALL, they fail to identify a genetic driver in a quarter of patients with childhood ALL and in over half of patients with adult ALL. Technological advances, particularly the introduction of next generation sequencing (NGS) with whole-exome, genome and transcriptome sequencing (RNA-seq), has dramatically increased our ability to detect these driver mutations. NGS can detect rearrangements not evident on conventional testing, such as the *DUX4*-rearranged ALL, or where these rearrangements converge onto a single gene [2].

Recently, a “revised taxonomy” of B-cell ALL (B-ALL) was described by Gu et al., consisting of 23 distinct genetic subtypes based on chromosomal rearrangements, somatic mutations and transcriptional profile [2]. RNA-seq was used to analyse leukaemic cells from patients with B-ALL to identify genetic alterations such as gene expression profiles, chromosomal rearrangements and alterations in copy number [2]. This greater understanding of the heterogenous drivers of ALL has not only resulted in enhanced risk stratification (Table 1) but provides the basis for developing new targeted therapies. A key example of this is “Philadelphia-like” ALL, discussed in detail later in this review, which carries both prognostic and therapeutic significance. 

NGS has also helped to identify driver mutations in T-ALL, however the prognostic impact of these subtypes and treatment implications are less well understood than in B-ALL. Studies in paediatric and young adult T-ALL have identified 106 putative driver mutations, with 10 recurrently mutated pathways and eight distinct molecular subgroups [3]. 

Studies in adult T-ALL have demonstrated that the presence of *NOTCH1* and/or *FBXW7* mutations in the absence of both *N*/*K-RAS* and *PTEN* mutations identifies a large patient group with a favourable outcome [4]. Conversely, the absence of *NOTCH1/FBXW7* or the presence of *RAS/PTEN* mutations identifies a poor risk sub-group. 

Early precursor T-cell ALL (ETP-ALL) is a recently identified subgroup of T-ALL. ETP-ALL is defined by a molecular profile and immunophenotype (CD1a–, CD8–, CD5weak with stem-cell or myeloid markers) consistent with maturation arrest at a very early stage of T cell differentiation, namely from a multipotent progenitor [5]. Importantly, ETP-ALL has been found to be associated with a poor prognosis, with patients experiencing very high rates of remission failure or relapse. 

A summary of current prognostic stratification of B and T-ALL based on cytogenetics/FISH and more recent molecular classification is shown in Table 1. As shown, precision approaches targeting patient-specific molecular vulnerabilities are emerging for a number of subtypes. However, in a significant proportion of patients, our increased understanding of the genetic and transcriptional aberration driving their disease has not yet been therapeutically exploited and further work is needed. 

## 3. The Power of Targeted Therapy: Philadelphia Chromosome Positive (Ph+) B ALL

The presence of the Philadelphia chromosome, a reciprocal translocation between chromosomes 9 and 22, t(9;22), results in the production of a BCR-ABL1 fusion protein product with constitutive tyrosine kinase activity. Ph+ ALL is a poor prognostic group in both childhood and adult ALL. 

The startling success of the tyrosine kinase inhibitor (TKI) imatinib in the treatment of chronic myeloid leukemia (CML) [18,19], also driven by the Philadelphia chromosome, overshadowed contemporaneous early studies demonstrating efficacy of imatinib in relapsed/refractory (R/R) Ph+ ALL [20,21]. However, TKIs have revolutionised the management and outlook of this disease. 

Studies have shown the combination of imatinib with chemotherapy is far superior to chemotherapy alone in patients with newly diagnosed Ph+ ALL [22,23,24,25,26,27,28], with CR rates of 90–95% and long-term survival of 40–50% [29,30,31]. The use of TKIs has become standard of care in the treatment of Ph+ ALL. In older patients, the addition of imatinib was shown to completely abrogate the negative prognostic impact of the Philadelphia chromosome [32]. However, allogeneic haematopoietic cell transplant (HCT) is still required to achieve a durable remission and is currently still recommended for eligible patients with a suitable donor. 

Given the activity of TKIs, the need for traditional chemotherapy in the management of Ph+ ALL is now under investigation. The GRAAPH-2005 trial demonstrated that imatinib combined with reduced intensity induction, dexamethasone plus vincristine, led to increased rates of complete remission compared with imatinib plus intensive chemotherapy with hyper-CVAD (cyclophosphamide/vincristine/doxorubicin/dexamethasone) (98% vs 91%; *p* = 0.006) [31]. This was primarily due to fewer induction deaths in the group receiving reduced intensity induction [31]. Achievement of molecular milestones was similar between the two groups with a trend towards improved event free survival (EFS) and overall survival (OS) in the reduced-intensity induction group.

The more potent second generation TKIs, dasatinib and nilotinib, have also been found to be highly effective in treating Ph+ ALL, arguably more so than imatinib. However, this has only been compared head-head in a phase 3 randomised control trial (RCT) in paediatric patients [33]. A trial by Foà et al. utilised the GIMEMA LAL1205 protocol, whereby 53 adult patients received dasatinib combined with steroids and no chemotherapy resulting in complete haematological remission in 92% of patients and a 20-month disease-free survival (DFS) and OS of 51.1% and 69.2%, respectively [34]. However, without post-consolidation treatment or allogeneic HCT, high rates of relapse were observed. 

In the follow-up GIMEMA LAL2116 D-ALBA trial, the combination of dasatinib and prednisolone was followed by two cycles of blinatumomab, an anti-CD19/CD3 bispecific T cell engager (BiTE). This resulted in a CR rate of 98% at the end of induction (day 84) with 29% of patients achieving a molecular response [35]. Following two cycles of blinatumomab, the proportional of patients achieving a molecular response increased to 60% [35]. With a median follow-up of 18 months, impressive results of 95% OS and 88% DFS were achieved with this chemotherapy-free dual targeted regimen [35]. 

Importantly, relapses following first or second generation TKIs are frequently associated with a BCR-ABL TKI-refractory threonine-to-isoleucine mutation at position 315 (T315I). In the phase 2 Ponatinib Ph+ ALL and CML Evaluation (PACE) trial, ponatinib, a third generation TKI with activity against unmutated and mutated BCR-ABL including the T315I mutation, demonstrated efficacy in R/R Ph+ ALL following failure of second generation TKIs [36]. The ponatinib starting dose was 45 mg once daily. In 32 patients with Ph+ ALL, a major haematologic response and major cytogenetic response were seen in 41% and 47%, respectively [36].

Ponatinib has subsequently been used upfront where the results of early non-comparative phase 2 studies suggest that it is more effective than earlier generation TKIs [37]. Currently, a phase 3 RCT (NCT03589326) comparing ponatinib and imatinib in combination with reduced-intensity chemotherapy in newly diagnosed Ph+ ALL in adults is underway. The results of this trial are eagerly awaited. 

Excitingly, given the deep responses seen with ponatinib, allogeneic HCT may no longer be indicated in patients achieving molecular milestones. It seems quite likely that in the not-so-distant future, Ph+ ALL will be managed with a chemotherapy-free targeted treatment approach. Such advances in Ph+ ALL and the use of TKIs in CML highlights the transformative potential of targeted therapy.

## 4. Philadelphia-Like B ALL

Ph-like or BCR-ABL-like ALL was first defined in paediatric patients with a gene expression pattern resembling BCR-ABL1 positive ALL but lacking the t(9;22) rearrangement and BCR-ABL1 fusion [38]. BCR-ABL-like ALL encompasses a subset of ALL with a diverse range of mutations. Notably, its definition and the range of tests performed to diagnose it varies between groups. Additionally, many centres may not have access to the range of assays, particularly gene expression microarray or RNA sequencing, required to diagnose BCR-ABL-like ALL.

Multiple studies have demonstrated that Ph-like ALL is associated with increased rates of treatment failure, particularly minimal residual disease (MRD) persistence, and poor survival outcomes [39,40]. Similar to BCR-ABL1 positive ALL, patients with BCR-ABL-like ALL have reduced five-year disease-free survival of 59.5% versus 84.4% for patients with other precursor B-ALL [38]. This was driven by an increased rate of relapse, 37% versus 16% [38]. Subsequently, it has been determined that the incidence of Ph-like ALL increases from 10–15% during childhood, through adolescence and reaching a peak in young adults where it represents 30% of disease. 

The underlying genetic alterations driving Ph-like ALL are heterogenous. However, these result in activation of common surface receptor/kinase signalling pathways: predominantly the JAK-STAT class (particularly via *CRLF2* rearrangements, such as *IGH*-*CRLF2* and *P2RY8-CRLF2*, which are seen in more than 40% of Ph-like ALL); less frequently ABL-class (*ABL1, ABL2, PDGFRA, PDGFRB, CSF1R*); and in smaller numbers RAS signalling (*KRAS, NRAS, PTPN11, CBL, NF1*) and other pathways (e.g., *FLT3, FGFR1, NTRK3*) [40,41]. 

Importantly, dependence on these signalling pathways provides the potential for therapeutic targeting. Defining the affected pathway and elucidating the specific targetable fusion is therefore essential. Pre-clinical studies have demonstrated activity of the JAK-inhibitor ruxolitinib and the TKI dasatinib in Ph-like murine xenograft models harbouring JAK-activating lesions or ABL-class fusions respectively. Synergy when combined with dexamethasone or phosphatidylinositol-3-kinase (PI3K)/mammalian target of rapamycin (mTOR) inhibition has also been seen [42,43,44]. Subsequent case reports have documented efficacy of ruxolitinib [45], imatinib [46,47], dasatinib [48,49] and ponatinib [50] in patients with refractory Ph-like ALL. Several clinical studies are now evaluating the efficacy of the addition of either ruxolitinib or TKIs in Ph-like ALL, and results are eagerly awaited (NCT03571321, NCT02420717, NCT02723994, NCT03117751, NCT02883049 NCT03564470, NCT02143414).

Furthermore, a recent case report has documented clinical activity of the tropomyosin receptor kinase (TRK) inhibitor lacrotrectinib in *ETV6-NTRK3* associated Ph-like ALL [51], and an active phase II clinical trial is evaluating this agent in TRK fusion R/R paediatric acute leukaemia (NCT03834961). 

## 5. Targeting the Menin-MLL1 Interaction in KMT2A Rearranged Leukaemia

The *KMT2A* (MLL) rearrangement at chromosome 11q23 is associated with poor prognosis in ALL (and AML), and is considered to be an indication for allogeneic transplant in eligible adult patients. MLL-fusion proteins resulting from these rearrangements bind to DNA/chromatin, resulting in aberrant gene expression and leukaemic transformation through interaction with chromatin-associated protein complexes. Targeting these critical interactions is an emerging strategy for the treatment of this disease. Menin, a KMT2A cofactor, is among the most promising of these. Preclinical studies have demonstrated clinical activity of oral small molecule Menin-MLL inhibitors in *KMT2A*-R cell line and murine models [52,53] and early phase clinical trials have recently been initiated in patients with R/R acute leukaemias. Enzymatic inhibitors of DOT1L, a histone 3 lysine 79 (K3K79) methyltransferase and essential downstream mediator of the KMT2A-R oncogenic program, is another promising approach [54,55].

**Figure 1:**
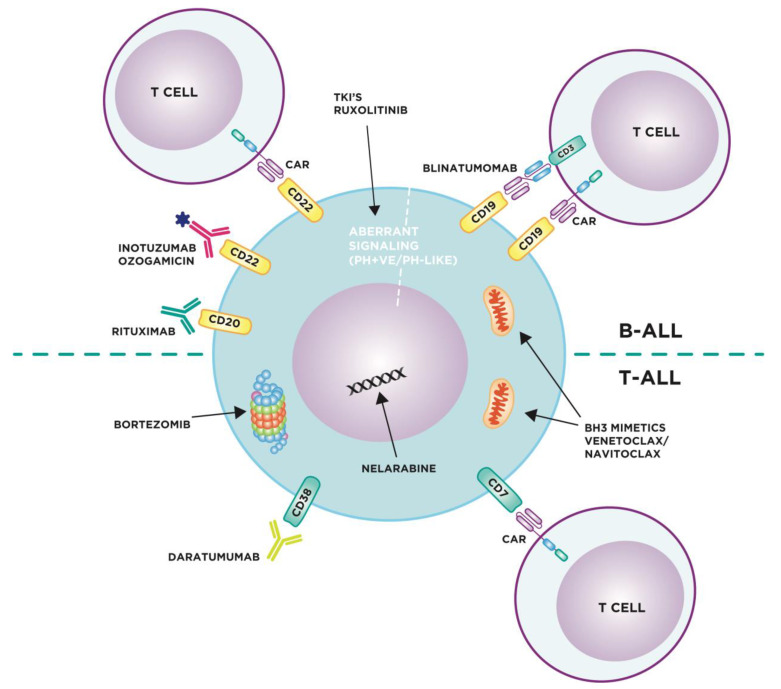
Targeted therapies in B- and T-ALL.

While personalised therapies exploiting disease-specific mutations are only possible in a subgroup of patients (Table 1), multiple current and emerging therapies target more general vulnerabilities in B- and T-ALL (Figure 1).

Depiction of targeted therapies in B- and T-ALL including: engagement of cytotoxic T cells either through a CAR T-cell directed against various cell surface receptors such as CD19, CD22 or CD7 or through the CD3/CD19 bispecific T-cell engager, blinatumomab; binding of monoclonal antibodies to induce antibody dependent cellular cytotoxicity and complement dependent cytotoxicity, or deliver a cytotoxic payload, via receptors such as CD22 (inotuzumab ozogamicin), CD20 (rituximab) or CD38 (daratumumab); blockade of aberrant cell proliferation signalling via JAK-activating lesions (ruxolitinib) or ABL-class fusions (tyrosine kinase inhibitors); induction of apoptosis via BH3 mimetics (venetoclax, navitoclax); inhibition of DNA synthesis in leukaemic blasts and induction of apoptosis by nelarabine; regulation of protein homeostasis by the proteasome inhibitor bortezomib.

CAR, chimeric antigen receptor (T cell therapy). TKI’s, tyrosine kinase inhibitors. PH + ve, Philadelphia chromosome positive ALL. PH-LIKE, Philadelphia chromosome-like ALL. 

## 6. Targeting the “B” in B-ALL

### 6.1. Targeting CD20

Surface expression of CD20, defined as expression in 20% or more of blasts, is seen in approximately 30% of B-ALL [56]. It has previously been found to be associated with an adverse prognosis [57]. However, this effect has not been consistent across studies and may depend on the specific chemotherapy regimen used.

Following its efficacy in chronic lymphocytic leukaemia (CLL) and B-cell non-Hodgkin lymphoma (NHL), the anti-CD20 monoclonal antibody rituximab was found to have clinical activity in combination with intensive chemotherapy in R/R B-ALL [58]. This is despite rituximab lacking single-agent activity in ALL [59]. Interestingly, chemotherapy and corticosteroids have been reported to upregulate CD20 surface expression, providing a potential biological explanation for greater efficacy when rituximab is combined with chemotherapy [60,61]. 

Subsequently, rituximab has been introduced into upfront therapy in combination with hyper-CVAD [62] and a paediatric ALL protocol [63], where it has been demonstrated to improve survival outcomes in adult patients younger than 60 years of age. Older patients did not derive the same benefit due to increased rates of infection and death in CR.

More recently, ofatumumab, an anti-CD20 monoclonal antibody with greater in vitro complement-dependent cytotoxicity than rituximab, has also been successfully combined with hyper-CVAD [64]. Obinutuzumab, a type II glycoengineered anti-CD20 monoclonal antibody, has shown activity in preclinical studies of ALL cell lines [65]. A clinical study comparing obinutuzumab versus rituximab in CD20 positive ALL patients is now underway (NCT04920968).

### 6.2. Blinatumomab

Blinatumomab is a bispecific anti-CD19/CD3 bispecific T cell engager (BiTE^®^) that has shown significant activity in the treatment of B-ALL. Blinatumomab is a fragment-based BiTE that simultaneously binds to CD19 positive B cells and the patient’s endogenous CD3 positive T cells. This induces T-cell engagement and antibody-dependent cellular cytotoxicity resulting in elimination of CD19 positive blasts. CD19 surface expression is present in more than 90% of B-ALL [56].

Blinatumomab is delivered as a continuous intravenous (IV) infusion for 28 days followed by 14 days off treatment. Efficacy of blinatumomab in R/R B-ALL was first demonstrated in a dose-finding study which was followed by a single-group multicentre phase 2 trial by Topp et al. [66,67]. In the phase 2 trial, 189 patients with primary refractory disease, relapse after allogeneic HCT or relapse after salvage chemotherapy were treated with blinatumomab. After two cycles of blinatumomab, 43% of patients achieved a CR or CR with partial haematological recovery [67]. The median overall survival was 6.1 months.

In the phase 3 TOWER study, 405 patients with R/R B ALL were evaluated. More than 50% of patients were in a second or later relapse. Patents were randomised 2:1 to receive blinatumomab or standard chemotherapy. Blinatumomab was associated with superior overall response rate (ORR) of 44% versus 25%, EFS (median 7.3 months versus 4.6 months) and OS (median 7.7 months versus 4.0 months) [68].

Importantly, patients with active central nervous system (CNS) or isolated extra-medullary disease were excluded from studies with blinatumomab and the efficacy of blinatumomab in these settings is uncertain. Intrathecal CNS prophylaxis is given during blinatumomab cycles, specifically before and after the blinatumomab infusion.

Blinatumomab is associated with reduced toxicity compared with chemotherapy. However, two specific toxicities are increased with blinatumomab: cytokine release syndrome (CRS) and neurological toxicity. In the TOWER study, the rate of grade 3 or higher CRS in patients treated with blinatumomab was 4.9%. Neurologic events that were grade 3 or higher occurred in 9.4% of blinatumomab-treated patients and in 8.3% of patients treated with chemotherapy [68].

Blinatumomab has also been studied in paediatric and young adult patients with R/R ALL. Similar efficacy and safety of blinatumomab has been demonstrated in heavily pre-treated paediatric and adolescent patients with R/R disease [69]. More recently, two large phase 3 RCTs, the COG AALL1331 and IntReALL HR2010 studies, have demonstrated superior efficacy and tolerability of blinatumomab following standard induction over high dose chemotherapy blocks in paediatric, adolescent and young adult patients with high or intermediate risk first relapse of B-ALL [70,71].

Persistence of measurable residual disease, detected by polymerase chain reaction (PCR), NGS or flow cytometry approaches, is the most important prognostic factor in both paediatric and adult ALL [72] and is predictive of relapse and reduced overall survival. Blinatumomab is currently the only therapy approved by the Food and Drug Administration (FDA) and equivalent regulatory bodies in non-US jurisdictions, for MRD positive ALL following intensive chemotherapy.

Approval followed results of a phase 2 single arm study by Gökbuget et al., which examined the use of blinatumomab in patients who remained MRD positive after at least three initial blocks of intensive chemotherapy. Eighty-eight (78%) of 113 patients converted to a MRD negative state. Patients who achieved MRD negativity also had a significantly improved median relapse-free survival (RFS), 23.6 versus 5.7 months, and OS, 38.9 versus 12.5 months [73].

A subsequent post-hoc analysis by Gökbuget et al. compared patients with MRD-positive B-ALL after initial chemotherapy who were treated with blinatumomab versus patients from a historic data set who received standard of care chemotherapy. Blinatumomab was associated with an improved median RFS (35.2 versus 8.3 months) and OS (36.5 versus 27.2 months) [74].

Multiple ongoing studies are now evaluating the role of blinatumomab in upfront therapy (NCT02003222, NCT03541083, NCT03914625, NCT03367299, NCT04307576, NCT03643276, NCT02877303, NCT03117751, NCT03480438, NCT03739814, NCT02744768, NCT04329325, NCT04530565, NCT04554485, NCT03523429, ACTRN12617000084381 and ACTRN12618001734257 studies).

### 6.3. Inotuzumab Ozogamicin

Surface expression of CD22 is seen in over 90% of cases of B-ALL with uniform expression in 80% and partial expression in 13% [56]. Inotuzumab ozogamicin (IO) is an anti-CD22 monoclonal antibody conjugated to calcheamicin, a cytotoxic agent. Upon binding to CD22 on the leukaemic blast, the CD22-conjugate complex is internalised, releasing calcheamicin which subsequently induces double-stranded DNA breaks thereby initiating apoptosis.

An initial phase 2 study by Kantarjian et al. demonstrated clinical activity of IO in R/R CD22 positive B-ALL [75]. Ninety patients were treated either with a single IV dose of 1.3 to 1.8 mg/m^2^ every three to four weeks or with a weekly dosing schedule. The ORR was 58% and the median survival was 6.2 months [75]. This trial established that weekly dosing resulted in similar efficacy with reduced toxicity compared to the single dose schedule.

Subsequently, the phase 3 INO-VATE ALL trial assessed 326 adults with CD22 positive R/R disease and compared IO with standard of care chemotherapy. This demonstrated superiority of IO, yielding higher rates of CR or CR with incomplete haematologic recovery (CRi) (73.8% versus 30.9%) and OS (median 7.7 months versus 6.2 months) [76,77]. In the initial 218 patients, higher rates of MRD negativity (78.4% versus 28.1%) and prolonged PFS (median 5.0 months versus 1.8 months) were seen in patients treated with IO [76].

There were significant differences in toxicity profile between IO and SOC chemotherapy highlighted in the INO-VATE ALL trial. While patients treated with IO had reduced frequency of grade 3 or more thrombocytopenia, transfusions, and grade 3 or higher febrile neutropenia, liver-related adverse events were more common. Specifically, veno-occlusive disease (VOD) occurred in 14% of patients receiving IO compared with 2.1% in the standard therapy group [77]. Subsequent allogeneic stem cell transplant and particularly use of a dual-alkylator conditioning regimen were key associated factors in patients developing VOD.

Strategies such as dose reduction and fractionation of IO, routine use of ursodeoxycholic acid and introducing a delay prior to allogeneic stem cell transplant conditioning, including bridging with subsequent blinatumomab, are now being employed to reduce the risk of VOD. Additionally, patients who achieve an MRD negative CR with IO are suggested to proceed to HCT after two cycles rather than receiving additional cycles due to the concern about VOD [78].

A current trial, NCT03677596, is testing if a lower dose of IO at 1.2 mg/m^2^ per cycle compared to 1.8 mg/m^2^ per cycle is safe and efficacious in patients at high risk of developing VOD.

The recent phase 2 ITCC-059 study has also demonstrated the efficacy of IO in 32 heavily pre-treated R/R paediatric and adolescent patients aged one to 17 years. Of the 28 treated patients, there was an ORR of 81.5%, including 95% achieving MRD negativity in those that responded, and 55% of patients were alive at one year [79]. Four cases of VOD/sinusoidal obstruction syndrome (SOS) were reported, including three of nine transplanted patients [79].

As with blinatumomab, a number of studies are now evaluating the role of inotuzumab ozogamicin in the upfront setting (NCT03150693, NCT04747912, NCT03959085, NCT03739814, NCT01371630, NCT02877303, NCT03249870, NCT03460522).

Other novel anti-CD22 monoclonal antibodies have been included in clinical trials but these have not demonstrated the response rates seen with IO. Anti-CD22 monoclonal antibodies including epratuzumab, moxetumomab pasudotox and the radioimmunoconjugate, 90Y-DOTA-epratuzumab, have been studied in R/R paediatric and adult ALL [80,81,82,83,84]. However, response rates with these constructs have been modest to date and their role in the treatment of ALL is currently uncertain.

## 7. Chimeric Antigen Receptor T Cell Therapy

Chimeric antigen receptor (CAR)-T cells are an exciting novel therapeutic approach in B-ALL. CAR-T cells are produced by taking the patient’s T cells or, less commonly, donor T cells, and then engineering them ex-vivo to express a synthetic receptor directed against a specific target antigen expressed on the cancer cell. T cells are subsequently infused into the patient following lymphodepletion.

CAR-T cells structurally consist of: an extracellular antigen binding domain, such as CD19 or CD22; a signalling domain, CD3zeta; and one or multiple co-stimulatory domains, such as 4-1BB and CD28. There is significant heterogeneity between research groups with regards to CAR-T cell design and production including the antigen targeted, binding affinity, co-stimulatory molecules used, manufacturing systems employed, transduction technique and source of T cells, autologous versus allogeneic. All of these factors influence the success of the CAR T-cell construct, which is dependent on availability of viable T cells, transduction efficiency, manufacturing time, T cell expansion, T cell persistence and efficiency of clearance of the leukaemic cells.

Tisagenlecleucel (tisa-cel), a second generation anti-CD19 CAR-T cell constructed with a CD3zeta signalling domain and 4-1BB co-stimulatory domain transduced into autologous patient T cells, was the first product approved by the FDA for the treatment of R/R B-ALL in patients up to 25 years of age. This approval was based on the results of the phase 2 ELIANA study. There were 75 patients aged 3 to 23 years. The median age was 11 years. Patients had received a median of three prior lines of therapy, including allogeneic HCT in 61% [85]. An impressive overall remission rate of 81% at three months was seen with all achieving MRD negativity [85]. The overall remission rate was defined as the rate of CR or CRi within three months. Persistence of the CAR-T cell was observed for as long as 20 months [85]. The 12-month EFS and OS in this heavily pre-treated population was 50% and 76% respectively [85]. The toxicity profile seen was similar to that of blinatumomab, though more pronounced, with 77% experiencing CRS, 47% grade 3–4, and 40% experiencing neurological toxicity, 13% grade 3 [85].

A criticism of CAR-T cell studies in general has been the reporting of outcomes only for patients who received their CAR-T cell infusion rather than the total enrolled study population. In the ELIANA study for instance, 18% (*n* = 17) of enrolled patients never received infusion due to tisa-cel product-related issues in seven patients, death prior to infusion in seven patients and adverse events in three patients [85]. Studies are now performing intention-to-treat analyses.

The role of tisa-cel is also being explored in the upfront setting. The Children’s Oncology Group (COG) AALL1721/CASSIOPEIA phase 2 single-arm study aims to determine the efficacy and safety of tisa-cel in de novo high risk paediatric and young adult B ALL patients who remain MRD positive at end of consolidation (NCT03876769).

The use of CAR-T cells in patients over the age of 25 years remains investigational. A phase 1 trial has been performed by Park et al., from the Memorial Sloan Kettering Cancer Center (MSKCC), of autologous T cells expressing the 19–28z CAR [86]. This CAR-T cell expresses a chimeric receptor with an anti-CD19 antibody binding site as well as intracellular domains from T-cell coactivating receptors, the CD3-zeta chain and CD28. This study examined 53 heavily treated R/R adult patients with a median age of 44 years where 68% of patients had received treatment with a third or later salvage. The CR rate was 83% with a median EFS and OS of 6.1 months and 12.9 months, respectively [86]. Among patients with low disease burden, namely less than 5% blasts thereby constituting MRD positive disease, the EFS and OS was 10.6 months and 20.1 months, respectively. Cytokine release syndrome occurred in 85% of patients with 26% experiencing grade 3 or higher. There was one death due to CRS. Grade 3 and 4 neurotoxicity were reported in 36% and 6% of patients, respectively.

Concerningly, the results of two recent studies suggest that prior blinatumomab may negatively impact response to anti-CD19 CAR-T cells in some patients. A single centre study by Pillai et al. examined 166 patients treated with tisa-cel, 16 of whom had received one to four cycles of blinatumomab prior to treatment with tisa-cel. Prior blinatumomab was associated with a significantly higher rate of failure to achieve MRD negative CR or subsequent loss of remission with antigen escape (*p* = 0.043) [87].

These results were confirmed in a larger multi-centre retrospective study by Taraseviciute et al. recently presented in abstract form. They identified 420 patients with R/R B-ALL below the age of 25 years who were treated with anti-CD19 CAR T-cell therapy [88]. Prior blinatumomab exposure, at a median of 169 days before CAR T-cell therapy, was reported in 75 patients. This was found to be associated with increased risk of CAR T-cell non-response, seen in 18.3% versus 7.0% (*p* = 0.0052), reduced EFS (5.8 versus 22.6 months) and increased incidence of negative or dimCD19 expression prior to CAR-T cell therapy (13.0% versus 6.2%) [88]. However, there was no difference in OS. Importantly, 10 out of 71 patients (14.1%) failed to respond to both CAR T-cell therapy and blinatumomab, suggesting an underlying resistance to CD19 targeting therapies [88]. However, blinatumomab non-response did not preclude CD19 CAR T-cell response, with 66% of blinatumomab non-responders still achieving remission with subsequent CD19 CAR T-cell therapy [88].

Sequencing of CD19 targeting agents is an emerging clinical conundrum. In light of the results of the phase 3 COG AALL1331 trial that showed superiority of blinatumomab compared to chemotherapy as post-reinduction therapy in first relapse of B-ALL in children and AYAs, as well as ongoing studies utilising blinatumomab in the upfront setting, a number of patients will potentially be exposed to blinatumomab before anti-CD19 CAR-T cell therapy [70]. Of note, prior blinatumomab was an exclusion criterion in the tisa-cel CAR T-cell ELIANA study.

Furthermore, antigen escape is an important mechanism of treatment failure with current immunotherapies in general. Dual targeting CAR-T cell strategies, such as CD19 and CD22, either as a single construct or a combination ‘cocktail’, are now being explored to potentially overcome this resistance pathway [89,90,91,92].

Natural killer (NK) cells are another type of immune cell that can kill tumour cells through cytotoxic mechanisms. Considering the success of CAR-T cell therapy, there is increasing interest in developing CAR-NK cell therapy. There are potential benefits to using CAR-NK cells including improved safety, with reduced cytokine release syndrome and neurotoxicity, and the possibility for an “off-the-shelf” product. Multiple groups have demonstrated pre-clinical activity of anti-CD19 CAR-NK cells in B-ALL cell lines, primary blasts and animal models [93,94,95]. Recently, cord-blood derived HLA-mismatched anti-CD19 CAR-NK cells have shown promising efficacy in patients with RR non-Hodgkin’s lymphoma or CLL [96]. Clinical trials utilising CAR-NK cells in ALL are now underway (NCT04796675, NCT03056339).

## 8. Targeting T-ALL

Unfortunately, while significant advances have been made in B-ALL in recent years, T-ALL has not seen the same progress and the prognosis for patients with high-risk disease such as the ETP subgroup, or patients who relapse, is poor. However, some important gains have been made and a number of emerging therapies are on the horizon.

### 8.1. Nelarabine

Nelarabine is a purine nucleoside analogue that is converted to 9-β-D-arabinofuranosylguanine (ara-G) which has been demonstrated to preferentially accumulate in T-lymphoblasts [97]. Two studies have examined single agent nelarabine in R/R adult T-ALL / lymphoblastic lymphoma (LBL): the Cancer and Leukemia Group B study 19801 by DeAngelo et al. and a phase 2 trial by Gökbuget et al. Single agent nelarabine led to a CR in 31% and 36% of patients, in the two trials respectively [98,99]. One-year overall survival was similar in the two trials at 28% and 24% [98,99].

A phase 2 trial by Berg et al. demonstrated nelarabine’s efficacy as well as establishing the tolerable dose to 650 mg/m^2^/day for 5 days in children [100]. Above this dose, toxicity such as peripheral neuropathy and somnolence was prominent.

Neurologic toxicity, especially in adults, has been an issue that has affected nelarabine’s use [101]. Strategies such as modifying the dose, schedule and using a continuous infusion may improve its tolerability. The German Multicenter Study Group for Adult ALL (GMALL) performed a prospective phase 2 trial in adults with R/R T-ALL/LBL and demonstrated that nelarabine given at 1500 mg/m^2^ days 1, 3 and 5 was tolerable. Neurotoxicity occurred in 16% of patients with 7% having grade 3 or 4 neurotoxicity [99]. These events included cognitive disturbance, confusion, Guillain–Barré-like syndrome, hallucinations and memory impairment.

In terms of haematological toxicities, nelarabine has a manageable toxicity profile even in heavily pre-treated patients. In the trial by DeAngelo et al., the main toxicities observed were grade 3–4 neutropenia (37% of patients) and grade 3–4 thrombocytopenia (26% of patients) [98].

More recently, nelarabine has successfully been incorporated into upfront therapy for paediatric, adolescent and young-adult patients. In the Children’s Oncology Group (COG) AALL0434 study by Dunsmore et al., 1562 patients with T-ALL aged one to 31 years were treated on an augmented Berlin-Frankfurt-Muenster (aBFM) regimen [102]. Patients were randomised to receive either high-dose MTX (HDMTX) with leucovorin rescue or escalating-dose methotrexate (MTX) without leucovorin rescue plus pegylated asparaginase (C-MTX). After induction, patients who were intermediate- or high-risk were also randomised to receive or not receive six 5-day courses of nelarabine incorporated into aBFM. Nelarabine was found to result in significantly improved 5-year DFS (88.2% versus 82.1%, *p* = 0.029) with similar toxicity, including neurotoxicity, between groups [102]. Importantly, CNS relapses were reduced in patients receiving nelarabine (1.3% versus 6.9%, *p* = 0.0001).

In the additional randomisation, Capizzi methotrexate (CMTX) escalating-dose methotrexate without leucovorin rescue plus pegylated asparaginase was found to be superior to high-dose methotrexate with leucovorin rescue (HDMTX). Patients who received both nelarabine plus CMTX had a 5-year disease free survival of 91% [102]. This is an interesting finding considering that HDMTX has been shown to be superior to C-MTX in high-risk B-ALL in children and adolescents. It has been stipulated that there may be a difference in sensitivity to methotrexate and asparaginase between B-ALL and T-ALL as well as noting the higher risk of CNS involvement or relapse in T-ALL. Furthermore, the findings in the AALL0434 study may also reflect differences in the dose of pegylated asparaginase, 6-mecaptopurine and the timing of cranial radiation therapy (CRT) between the groups.

In adult patients with T-ALL/LBL, although the addition of nelarabine to hyper-CVAD in a single-centre phase 2 study by Abaza et al. was well tolerated, it was not associated with a survival benefit. Nelarabine with hyper-CVAD was compared to historical controls receiving hyper-CVAD alone and the three-year CR duration and OS rates were 66% and 65%, respectively [103].

A follow-up study is currently recruiting with earlier introduction of nelarabine and addition of pegylated-asparaginase, with promising early data recently presented by Maiti et al. [104].

### 8.2. Other T Cell Targeting Therapies

Bortezomib is a proteasome inhibitor routinely used in the management of multiple myeloma that has demonstrated activity in ALL, particularly T-ALL but also in pre-B ALL, in combination with chemotherapy. In the phase 1 study, T2005-003, bortezomib with chemotherapy demonstrated an ORR of 73% in 22 patients with relapsed ALL, 20 of whom had B-precursor ALL [105].

The Children’s Oncology Group demonstrated an encouraging second complete remission (CR2) rate of 68% +/- 10% in patients with high-risk first relapse of T-ALL [106], which compared favourably to limited available historical data. Three-year PFS and OS were 75% and 67%, respectively, for patients who achieved MRD negativity (<0.01%) versus 43% and 44%, respectively, for patients who remained MRD positive after one cycle [106].

Bortezomib has subsequently been evaluated in upfront therapy in paediatric and AYA T-ALL/LBL combined with an aBFM backbone in the COG AALL1231 phase 3 RCT [107]. This study planned to recruit 1400 patients aged one to 30 years. However, this study was closed early after recruiting 823 patients following the results of the aforementioned AALL0434 study that demonstrated superiority of nelarabine which was not included in this protocol.

In the COG AALL1231 trial, cranial radiation was omitted for all except very high risk (VHR) patients. In the context of this reduced sample size, the addition of bortezomib was associated with a trend toward improved three-year EFS (85.1% versus 81.7%, *p* = 0.0736) and OS (88.2% versus 85.5%, *p* = 0.087) but failed to reach statistical significance [107].

Standard (SR) and intermediate risk (IR) patients as well as patients with T-lymphoblastic lymphoma (LBL) were demonstrated to have superior outcomes with bortezomib compared to those not given bortezomib. EFS in patients who received bortezomib compared to those that did not was 92.5% versus 85.1% (*p* = 0.046) in SR patients; 90.3% versus 85.9% (*p* = 0.010) in IR patients; and 88.3% versus 76.5% (*p* = 0.007) in patients with T-LBL [107]. Conversely, the outcomes for VHR patients were dismal and worse with bortezomib with an EFS of 6.5% versus 37.5% (*p* = 0.038) [107].

## 9. T Cell Targeted Immunotherapy

While targeted immunotherapy has had a dramatic impact on the treatment landscape and outcomes for patients with B-ALL, this has not been the case for T-ALL. There are not currently any approved immune/surface protein targeting therapies for T-ALL. However, multiple approaches are currently being explored.

CD38 has been shown to be robustly expressed on T-ALL blasts in patient samples [108,109]. Preclinical studies have demonstrated efficacy in patient derived xenograft models with daratumumab, an anti-CD38 targeting monoclonal antibody which is FDA approved in multiple myeloma [109]. Several case reports have subsequently documented response to daratumumab in patients with relapsed/refractory T-ALL [110,111,112].

CAR-T cell strategies are clearly more difficult in T-ALL with very limited options for “pan” T-ALL specific antigenic targeting. There are significant obstacles including CAR-T cell fratricide and prolonged host T cell aplasia. One of the more promising targets is CD7, a pan T cell antigen expressed on normal and malignant T cells, including approximately 95% of T-ALL. A number of strategies have been utilised to avoid CAR-T cell fratricide. These include genomic disruption of CD7 and the use of a nanobody to retain CD7 within the endoplasmic reticulum (ER) and Golgi thereby preventing its surface expression [113,114,115]. The early results of single-arm studies appear promising and further data is eagerly awaited.

CD1a, another novel target being explored for CAR-T cells in T-ALL, is specifically expressed in cortical thymocytes and in about 40% of T-ALL cases. Pre-clinical studies have demonstrated robust cytotoxicity both in vitro and in vivo in xenograft models while avoiding the issue of fratricide and sparing mature T cells [116]. While this approach holds promise, it will only be relevant for a minority of patients with T-ALL with relapsed CD1a+ disease and likely mechanisms of escape will be loss of CD1a expression or selection of a pre-existing CD1a- subclone.

## 10. Targeting Intrinsic Apoptosis in ALL

Cell intrinsic apoptosis is regulated by the complex interaction of three layers of BCL-2 family-member proteins: the pro-apoptotic BH3-only proteins, BIM, BMF, PUMA, NOXA, BIK, BID, BAD and HRK, inhibit the pro-survival proteins in response to cellular damage; the pro-survival BCL-2 family proteins, BCL-2, BCL-XL, BCL-W, MCL-1, and A1, antagonise the effectors of apoptosis; and downstream effectors of apoptosis, BAX and BAK [85,117].

BH3-mimetics, a novel class of therapeutics, inhibit the action of specific pro-survival BH3-only proteins to induce apoptosis. Importantly, cancer cells are usually dependent on one or more specific BH3-only protein for their survival, governed by their underlying transcriptional profile which is driven by both cell of origin and specific oncogenic drivers.

Venetoclax, a specific BCL-2 inhibitor, is a first-in-class BH3-mimetic that is FDA approved in both CLL and acute myeloid leukaemia (AML). Preclinical studies have demonstrated dependence on BCL-2 in a significant proportion of both ALL cell lines and patient xenograft models [7,118]. However, in contrast to CLL, effective anti-leukaemic activity in ALL frequently requires dual inhibition of BCL-2 and BCL-XL [119].

A number of studies in both adult and paediatric R/R ALL are underway, evaluating the efficacy of venetoclax in combination with chemotherapy, and/or navitoclax, a BCL-XL and BCL-2 inhibitor. The rationale for this latter combination is to minimise the dose-limiting thrombocytopenia seen with single agent navitoclax whilst enhancing the synergistic effect of dual BH3-only protein inhibition.

Early data for venetoclax with navitoclax are promising. A preliminary report of 36 heavily pre-treated adult patients treated with venetoclax plus navitoclax with or without chemotherapy demonstrated good tolerability with CR, CRi or CR with incomplete platelet recovery (CRp) achieved in 20 out of 36 (56%) patients [120]. In the patients who achieved CR, CRi or CRp, 10 out of 18 patients (56%) had undetectable MRD [120].

A similar phase 1 study in 18 heavily pre-treated paediatric patients with R/R disease demonstrated similar tolerability and efficacy. Ten out of 18 patients (56%) achieved a CR, CRi or CRp, with MRD negativity in 70% of responders [121].

As described above, ETP-ALL is a recently identified subgroup associated with a poor prognosis with standard therapy. BH3-profiling of cell lines and patient samples has demonstrated that while T-ALL is primarily dependent on BCL-XL, ETP-ALL is dependent instead on BCL-2 with in vitro and in vivo sensitivity to BCL-2 inhibition [122]. Excitingly, this correlated with the results of early clinical studies with responses, including achievement of MRD negativity, documented in patients with ETP-ALL treated with venetoclax in combination with navitoclax or chemotherapy [120,121,123].

## 11. Targeting Chemokines and Their Receptors in ALL

The bone marrow tumour microenvironment is important in ALL, as leukaemic cells can disrupt normal haematopoietic function, promote their own proliferation and find protection from the effects of chemotherapy. ALL cells reside with mesenchymal stromal cells (MSCs) in this niche. The homing of ALL cells to this bone marrow microenvironment is postulated to be akin to that of haematopoietic stem cells (HSCs). Stromal cell-derived factor 1 (SDF1/CXCL12) is a chemoattractant produced by MSCs which binds to its receptor, CXCR4 [124]. Disruption of this CXCR4/CXCL12 axis leads to HSCs migrating to the peripheral blood. This may be a relevant target in ALL.

In ALL, there is a high level of surface CXCR4 expression and disrupting the CXCR4/CXCL12 axis decreases ALL engraftment in animal models [124]. As such, early phase clinical trials (NCT02763384 and NCT01319864) are trialling the use of CXCR4 inhibitors such as plerixafor and BL-8040 in relapsed ALL. These agents are being used in combination with other agents as they aim to increase the sensitivity to agents by mobilising leukaemic blasts into the peripheral blood.

Other cytokines and their receptors are also important as there are CXCR4/CXCL12-independent mechanisms attracting leukaemic cells to the tumour microenvironment. CCL25/CCR9 and CCR5 are two other potential targets where agents are in early development.

## 12. Epigenetic Regulators in ALL

Epigenetic modulation of oncogenes drives transcription in many types of cancers including certain subtypes of ALL. Several classes of drugs act at the epigenetic level including histone deacetylase (HDAC) inhibitors and inhibitors of bromodomain and extra-terminal motif (BET) proteins.

BET proteins are recruited to chromatin via their bromodomains and then activate and increase the rate of transcription of oncogenes such as *MYC*. These proteins have an important role in cell survival and therefore several BET inhibitors have entered clinical trials. In ALL, BET inhibitors have shown efficacy in arresting growth of MLL-AF4^+^ leukaemic cells and in combination with imatinib in NUP214-ABL1/TLX3-expressing T-ALL cells [125,126].

However, BET inhibitors have shown only limited efficacy in other diseases such as acute myeloid leukaemia and thrombocytopenia and gastrointestinal toxicity have limited their use [127]. Therefore, it will be important to identify the patient subsets with ALL where BET inhibitors may be efficacious as well as using them in combination with agents such as HDAC inhibitors so that they may be used at lower doses to avoid toxicity.

Histone deacetylases are enzymes that regulate gene expression through removal of acetyl groups from proteins including histones [128]. HDAC inhibitors also reduce Myc expression and thereby mitigating its oncogenic effect [128]. In certain types of T-ALL cell lines, such as Notch-driven T-ALL, panobinostat has been shown to induce apoptosis as well as downregulating *Myc* [129]. However, a phase 1 trial of the oral HDAC inhibitor abexinostat, including patients with ALL, showed limited efficacy as a single agent [130]. Combination therapies where an HDAC inhibitor is combined with cytotoxic agents, such as cytarabine, may be more effective as shown in *KMT2A*-rearranged ALL murine models [131].

## 13. Challenges of Clonal Heterogeneity and Leukaemia Initiating Cells (LICs)

There is increasing appreciation of the underlying clonal heterogeneity of ALL and its impact on both disease emergence and evolution, particularly in the context of treatment-induced selective pressures [132,133]. The presence of genetic and phenotypic diversity within leukaemic subclones has important implications for the success of targeted therapy. Lack of dependence on a therapeutically exploited pathway or absence of expression of a target antigen will result in treatment failure. This potentially can be overcome through both a greater understanding of this variance within the cancer population through single cell technologies and through the use of combination approaches.

Adding further complexity, a number of groups have reported the identification in B- and T-ALL of leukaemia-initiating cells (LICs), a rare distinct stem cell-like population which have self-renewal capacity, and the ability to differentiate into the leukaemic cells that make up the majority of the tumour bulk [134,135,136,137]. These have generally been defined by their capacity to initiate leukaemia in xenograft mouse models. While there remains significant controversy in this area, there is emerging evidence that LICs may be inherently chemotherapy and glucocorticoid resistant [138] and demonstrate significant plasticity [139]. Evaluation of rational targeted and combinational strategies to target LICs are currently being evaluated in pre-clinical studies [139,140].

## 14. Conclusions

Traditional chemotherapy has run its course in the treatment of ALL and further significant gains are unlikely to be made through protocol optimisation alone. The use of novel targeted approaches has already been demonstrated to be superior in relapsed disease to chemotherapy alone. Emerging data also supports their incorporation into upfront therapy.

This is not to dismiss the importance of current chemotherapy containing treatment backbones which have been honed through years of rigorous clinical trial methodology. Paediatric ALL has demonstrated the outcomes that can be achieved with intensive chemotherapy where very good cure rates are seen, albeit at the expense of long-term survivorship issues for many patients.

Asparaginase or pegylated-asparaginase, which could also be considered a targeted therapy, is an essential component of current ALL therapy in paediatric and AYA patients, with significantly worse outcomes reported if patients are unable to complete their intended treatment course per protocol [141].

In the immediate future, targeted therapies will be combined with chemotherapy rather than replacing it. In upfront disease this will see targeted therapies replacing intensive chemotherapy blocks with the aim to increase depth of response whilst reducing toxicity. Two major exceptions are, firstly, Ph+ ALL, where the use of combinational targeted therapies such as ponatinib and blinatumomab could obviate the need for chemotherapy. Secondly, in elderly patients who suffer increased morbidity and mortality with intensive chemotherapy, targeted therapies reduce exposure to cytotoxic chemotherapies and the risks that they bring.

Sequencing and rational combination of targeted agents are important questions that need answering in current and future clinical studies. Important clinical issues and questions already encountered through the increasingly complex treatment landscape include the use of blinatumomab prior to anti-CD19 CAR-T cell therapy or planning for allogeneic stem cell transplant following inotuzumab ozogamicin and managing the risk of VOD.

As our understanding of the disease and our armamentarium continues to evolve, there is increasing optimism in the treatment of ALL. There are significant gains still to be made, particularly in management of high-risk disease including T-ALL. However, there is a sense that we are at the start of a treatment revolution.

## Figures and Tables

**Table 1:** Prognostic stratification in ALL based on standard cytogenetics/FISH and more recent molecular classification.

Genetic Subtype	Prevalence [2,4,5]	Genomic Alteration	Prognosis [2,4,5]	Diagnostic Assay	Opportunities For Targeted Therapy
**B-ALL**					
High hyperdiploid (>50 chromosomes)	Children (25%) >> AYA > Adult	Ras pathway	Favourable	Cytogenetics-FISH	
*ETV6-RUNX1* t(12;21)(q13;q22)	Children (30%) >> AYA = Adult	*ETV6-RUNX1, PAX5* deletion, *WHSC1* mutation	Favourable	Cytogenetics-FISH, RT-PCR	
*DUX4*-rearranged	Children < AYA (~8%) > Adult	*DUX4*-rearranged, *ERG* deletions	Favourable	NGS approaches (cryptic)	
*TCF3-PBX1* translocation t(1;19)(q23;p13)	Children = AYA = Adult (5%)	*TCF3-PBX1*	Favourable with modern intensive (CNS directed) therapy	Cytogenetics-FISH	
*NUTM1* rearrangement	Children only, very rare	Most common partner is *BRD4*	Favourable	FISH (also NGS approaches)	Theoretical role for BET inhibitors and HDAC inhibitors [6].
Near haploid (24–30 chromosomes)	Rare (<3%) children, AYA and adults	*IKZF3* deletions, Ras pathway	Intermediate	Cytogenetics-FISH	Preclinical data demonstrating activity of BCL2 inhibition [7].
*ZNF384*-rearranged	Children < AYA (5–10%) > Adult	Fusions involving transcriptional regulators/chromatin modifiers	Intermediate	NGS approaches (predominantly RNA sequencing)	Preclinical activity of HDAC inhibitors in leukaemic cells harbouring ZNF384-EP300 or -CREBP fusions [8].
*PAX5alt*	Children (11%) > AYA/Adults	*PAX5* fusion, mutation, amplification	Intermediate	NGS approaches (predominantly RNA sequency)	
*PAX5* P80R	Children< AYA < Adults (~4%)	*PAX5* mutation, Ras pathway, *JAK-STAT* pathway	Intermediate in children. Intermediate -favourable in adults.	NGS approaches	Theoretical role for JAK inhibitors.
Low hypodiploid (31–39 chromosomes)	Children < AYA < Adults (10–15%)	*TP53* mutation, *IKZF2* deletion	Poor	Cytogenetics-FISH	Preclinical data demonstrating activity of BCL2 inhibition [7].
*BCR-ABL1* t(9;22)(q34;q11.2) Philadelphia (Ph) chromosome	Children < AYA < Adults (>25%)	*BCR-ABL* fusion, deletions of *IKZF1*, *CDKN2A/2B* and *PAX5*	Poor (Improved with TKIs)	Cytogenetics-FISH, RT-PCR	TKIs standard in frontline therapy.
Ph-Like	Children < AYA (25–30%) > Adults	Multiple kinase alterations (see text), *CRLF2* rearrangement, *IKZF1* deletion.	Poor	Complex. Multiple testing algorithms exist [9,10]. Flow cytometry (CRLF2), FISH, targeted PCR panels and LDA used for screening. RNA sequencing aids identification of potentially treatable fusions.	JAK inhibitors (JAK-STAT class), TKIs (ABL1 class) currently in clinical trials for upfront therapy. Clinical study of TRK inhibitor, lacrotrectinib, in R/R paediatric ALL with TRK fusion (NCT03834961).
*MEF2D* rearrangement	Children < AYA (~7%) > Adult	Ras pathway, *HDAC9* activation.	Poor	NGS approaches (predominantly RNA sequencing)	Preclinical evidence for HDAC inhibitors [11].
iAMP21	Children = AYA > Adult	Intrachromosomal amplification of chromosome 21. *RUNX1* amplification, *RB1* and *EBF1* deletion. Ras pathway mutations.	Poor	FISH	Preclinical evidence for MEK1/2 inhibitor selumetinib [12].
*IGH* rearrangement	Children < AYA (11%) > Adult	Partner genes include *CRLF2, CEBP* family, *ID4, BCL2, MYC, BCL6*.	Poor overall. Prognosis depends on specific partner gene involved.	FISH. NGS approaches to define partner genes	
*TCF3-HLF* t(17;19)(q22;p13)	Rare (<1%)		Very poor	Cytogenetics-FISH	Activity of BCL2 inhibition in patient derived xenografts [13].
*KMT2A* rearranged	Infants >> Children < AYA < Adult (15%)	Ras pathway	Very poor	Cytogenetics-FISH	Menin-MLL inhibitors now in clinical studies, pre-clinical activity of DOT1L inhibitors (see text).
**T-ALL**					
*NOTCH1* and/or *FBXW7* mutation and wild type *RAS*/*PTEN*	~50% T-ALL	Notch1 pathway	Favourable	Targeted sequencing/NGS approaches	Promising preclinical activity of gamma secrectase inhibitors (GSI) not borne out in initial clinical studies and dose limiting GI toxicity seen [14]. Combination treatment with dexamethasone (NCT02518113, NCT01363817) and selective GSI strategies now being evaluated [15].
*NOTCH1* and *FBXW7* wild type	~30% Adult T-ALL		Poor	Targeted sequencing/NGS approaches	
*RAS* or *PTEN* mutation/deletion	~20% Adult T-ALL	Ras pathway	Poor	NGS approaches	
ETP-ALL	10–15% T-ALL	See text	Poor. However, prognosis may be improved with modern MRD directed treatment strategies [16,17].	Initially defined by gene expression profiling. However, more commonly now by flow cytometry.	Pre-clinical and early clinical suggestion of responses to venetoclax (see text).

AYA, adolescent and young adult. TKIs, tyrosine kinase inhibitors. FISH, fluorescence in situ hybridization. NGS, next generation sequencing. MRD, measurable residual disease. CNS, central nervous system. BET, bromodomain and extra-terminal. HDAC, histone deacetylase. LDA, low density microarray. TRK, tropomyosin receptor kinase. ETP-ALL, early precursor T-cell ALL. R/R, relapsed refractory.
